# Effect of short-term compression therapy after thermal ablation for varicose veins: study protocol for a prospective, multicenter, non-inferiority, randomized controlled trial

**DOI:** 10.1186/s13063-023-07609-1

**Published:** 2023-10-12

**Authors:** Mingjun Tang, Weihua Jiang, Jin Hong, Lubing Li, Dan Shang, Yue Zhao, Zhenjie Liu, Ming Qi, Mingjuan Jin, Yuefeng Zhu

**Affiliations:** 1 Department of Vascular Surgery, The Fourth Affiliated Hospital, Zhejiang University School of Medicine, Yiwu, China; 2 International Institutes of Medicine, Zhejiang University, Yiwu, China; 3 Jiangsu Province Hospital of Traditional Chinese Medicine, Nanjing, China; 4 Affiliated Hospital of Shaoxing University, Shaoxing, China; 5 Yantai Yuhuangding Hospital, Yantai, China; 6 Union Hospital, Tongji Medical College, Huazhong University of Science and Technology, Wuhan, China; 7 China-Japan Union Hospital of Jilin University, Changchun, China; 8 The Second Affiliated Hospital of Zhejiang University School of Medicine, Hangzhou, China; 9 The First Affiliated Hospital of Dalian Medical University, Dalian, China; 10 School of Public Health, Medical School of Zhejiang University, Hangzhou, China; 11 Department of Vascular Surgery, Sir Run Run Shaw Hospital, Hangzhou, China

**Keywords:** Varicose veins, Thermal ablation, Compression therapy, Targeted vein occlusion rate, Randomized controlled trial, Study protocol

## Abstract

**Background:**

For patients with varicose veins, the goal is to relieve pain and swelling, reduce the severity of edema, improve skin changes, and heal ulcers associated with venous disease. Compression therapy is the cornerstone of their management. Several studies have shown that wearing an elastic bandage for the first 24 h and then a compression stocking for a week can effectively reduce the pain after thermal ablation. However, in clinical practice, patient compliance with this treatment could be better, considering difficulties in pulling up and removing the compression stocking, tightness, and skin irritation because these must be worn for a prolonged period. A potential solution to battling these barriers is short-term compression therapy. Besides, the effect and necessity of wearing compression stockings after thermal ablation have been questioned. Based on current clinical experience and limited evidence, although some scholars have suggested that compression therapy may be an unnecessary adjunctive therapy after thermal ablation, there is still a great deal of uncertainty in the absence of compression therapy after thermal ablation compared to compression therapy. Therefore, we advocate further research to evaluate the clinical effect of short-term postoperative compression therapy. Furthermore, well-designed randomized controlled trials are needed.

**Methods:**

A prospective, multicenter, non-inferiority randomized controlled trial is designed to evaluate the non-inferiority of target vein occlusion rate at 3 months. Three hundred and sixty patients will be randomly assigned in a 1:1 ratio to one of the following treatments: (A) 3 M™ Coban™ elastic bandage for 48 h or (B) 3 M™ Coban™ elastic bandage for the first 24 h and then a class II compression full-length stocking (23–32 mm Hg) for 1 week. The two groups will be compared on several variables, including target vein occlusion rate at 3 months (primary outcome indicator), pain, quality of life, clinical severity of varicose veins, postoperative complications, time to return to regular work, and compliance.

**Discussion:**

Suppose the effect of the 3 M™ Coban™ elastic bandage for 48 h proves to be non-inferior to long-term compression therapy. In that case, this short-term treatment may contribute to a future update of clinical guidelines for compression therapy after thermal ablation of varicose veins, resulting in higher patient compliance and better postoperative quality of life.

**Trial registration:**

Clinical Trials NCT05840991. Registered on May 2023.

**Supplementary Information:**

The online version contains supplementary material available at 10.1186/s13063-023-07609-1.

## Introduction

### Background

*Varicose veins* are a disease/syndrome in which the subcutaneous veins of the lower extremities are dilated, prolonged, and tortuous, reaching at least 3 mm in diameter[1]. Varicose veins are present in 10–30% of the general population; this percentage increases with age [2]. The conventional treatment for varicose veins is high ligating/stripping (HL/S) of the great saphenous vein [3]. Since the 1990s, radiofrequency ablation (RFA), endovenous laser ablation (EVLA), and ultrasound-guided foam sclerotherapy (UGFS) have been widely used in the treatment of varicose veins [4]. Among them, RFA and EVLA have primarily replaced traditional surgery with shorter operative time, lower postoperative complication rates, and shorter recovery times [5–13]. Compression therapy is commonly used after invasive treatment of varicose veins in the lower extremities, a strategy inherited from post-surgical varicose vein care. It aims to maintain occlusion of the treated veins and prevent bruising and recanalization [14]. Elastic bandages and compression stockings are the most popular form of compression devices.

The American Venous Forum suggests starting with an elastic bandage a few days after the procedure, and the pressure should be over 20 mmHg [15]. Based on the experience of generations of clinicians and relevant evidence-based medical evidence, the National Institute of Health and Care Excellence recommends using compression (bandaging or hosiery) for no more than 7 days after interventional treatment for varicose veins [16]. Several studies have shown that wearing an elastic bandage for the first 24 h and then a compression stocking for a week can effectively reduce the pain after thermal ablation. Elderman [17] compared the difference in pain between those who wore elastic bandages for the first 24 h and then a class II compression stocking for 2 weeks and those who only used elastic bandages for 24 h. It was found that compression stocking was significant for pain relief during the first week postoperatively. Ye [18] also compared the above compression therapies and found that wearing compression stockings reduced the severity of pain during the first postoperative week in C2 varicose veins patients. However, there was no significant benefit after wearing the stockings for more than 1 week. Based on this, Bootun [19] compared the outcomes of patients treated with compression stockings for 1 week after an elastic bandage for the first 24 h with those who only wore an elastic bandage for 24 h. They found that patients in the compression stockings group had significantly lower pain scores than others at 2–5 days; it suggests that wearing compression stockings for a week after venous thermal ablation may be sufficient to relieve pain. However, poor patient compliance is a frequent problem in clinical practice with wearing compression stockings, with considerations related to difficulties in pulling up and removing the compression stockings, concerns with tightness, and skin irritation because these must be worn for a prolonged period [20]. In addition, subsequent clinical trials have questioned the necessity and effect of wearing compression stockings after thermal ablation. AYO [21], who evaluated whether wearing stockings for 1 week after endovenous thermal ablation could improve clinical outcomes in patients, showed that compression therapy had no significant effect on the outcome after thermal ablation in patients with non-ulcerated varicose veins. Pihlaja [22] found that patients without compression therapy after RFA had a 100% vein occlusion rate at 6 months compared to those wearing compression stockings for 2 weeks. The study results of Onwudike [23] also showed that the clinical effect of not wearing compression socks after thermal ablation was not worse than that of wearing compression socks for 2 weeks, which supported the conclusion that the extensive use of compression socks for compression therapy after thermal ablation had no clinical benefits for patients. Besides, a meta-analysis suggested that 48 h may be an ideal short-term compression therapy for patients with varicose veins treated with thermal ablation only [24].

Based on current clinical experience and limited evidence, although some scholars have suggested that compression therapy may be an unnecessary adjunctive therapy after thermal ablation, there is still a great deal of uncertainty in the absence of compression therapy after thermal ablation compared to compression therapy. Therefore, we advocate further research to evaluate the clinical effect of short-term postoperative compression therapy. In summary, the quality of current published studies must be improved due to the small sample size, lack of detailed data on compression therapy, and inconsistent findings. Moreover, most existing studies focus on postoperative pain, with less attention to the target vein occlusion rate; further well-structured randomized controlled trials are needed. In this study protocol, we propose to evaluate the non-inferiority of the target vein occlusion rate at 3 months. We expect to provide a basis for simplifying the compression therapy after thermal ablation.

### Objectives

The study’s primary objective is determining the target vein occlusion rate at 3 months, assessed with a Duplex ultrasound scan. Recanalization will be defined by a segment of the vein ≥ 5 cm. Secondary objectives are to compare the two treatment groups for pain scores at baseline, 1 week, and 3 months using a visual analog scale (VAS); quality of life scores at baseline, 1 week, and 3 months using the AVVQ (Aberdeen Varicose Vein Questionnaire); clinical change using the VCSS (Venous Clinical Severity Score) at baseline, 1 week, and 3 months; postoperative complications including edema, ecchymosis, paresthesia, skin burn/discoloration, phlebitis, hematoma, infection, deep vein thrombosis, and pulmonary embolism; time is taken to return to routine work; patient compliance with the intervention assessed with a questionnaire.

### Methods

The following information follows the SPIRIT reporting guidelines [25].

#### Study design

The study design is a prospective, multicenter, non-inferiority, randomized controlled trial.

#### Study setting

The current study will take place in nine hospitals in six provinces in China. The centers of this study are as follows:

- The Fourth Affiliated Hospital of Zhejiang University School of Medicine
- Sir Run Run Shaw Hospital of Zhejiang University School of Medicine
- The Second Affiliated Hospital of Zhejiang University School of Medicine
- Union Hospital, Tongji Medical College, Huazhong University of Science and Technology, Wuhan
- China-Japan Union Hospital of Jilin University
- Yantai Yuhuangding Hospital
- The First Affiliated Hospital of Dalian Medical University
- Affiliated Hospital of Shaoxing University
- Jiangsu Province Hospital of Traditional Chinese Medicine

#### Participants

This trial was conducted in patients with primary varicose great saphenous veins of lower extremities diagnosed in nine target hospitals from April 2023 to August 2023. All participants included in the trial signed an informed consent form.

#### Inclusion criteria

The inclusion criteria are aged 18 to 80, primary unilateral lower limb saphenous varicose veins; clinical grades C2 to C4; diameter of the main trunk of the saphenous vein in the thigh segment ≥ 2 mm to ≤ 15 mm; and agree to participate in this study and voluntarily sign the informed consent form.

#### Exclusion criteria

The exclusion criteria are known to have difficulty tolerating surgery; the main trunk of the saphenous vein is extremely twisted or tightly attached to the skin; previous history of ipsilateral varicose vein surgery in the lower extremity; combined deep vein thrombosis or previous deep vein thrombosis in the affected lower extremity; uncorrectable coagulation dysfunction or a significant blood abnormalities with significant bleeding tendency (platelets ≤ 30 × 10^9/L); ankle-brachial index (ABI) < 0.6 and/or absolute ankle pressure < 60 mmHg; known allergy to bandage or stocking used in the treatment; pregnancy or breastfeeding; involvement in other clinical trials; and other patients deemed unsuitable for this study by the investigator.

#### Randomize and blinding

Patients will be randomized (using a random number table generated by the Excel RANDOM function) to group A or B. A fixed data analyst will generate the table of random numbers via computer. The randomization scheme will be hidden from doctors and patients. Since the intervention in this trial could not blind the doctors and patients, only the data analyzers were blinded.

#### Description of intervention

**Fig. 1:**
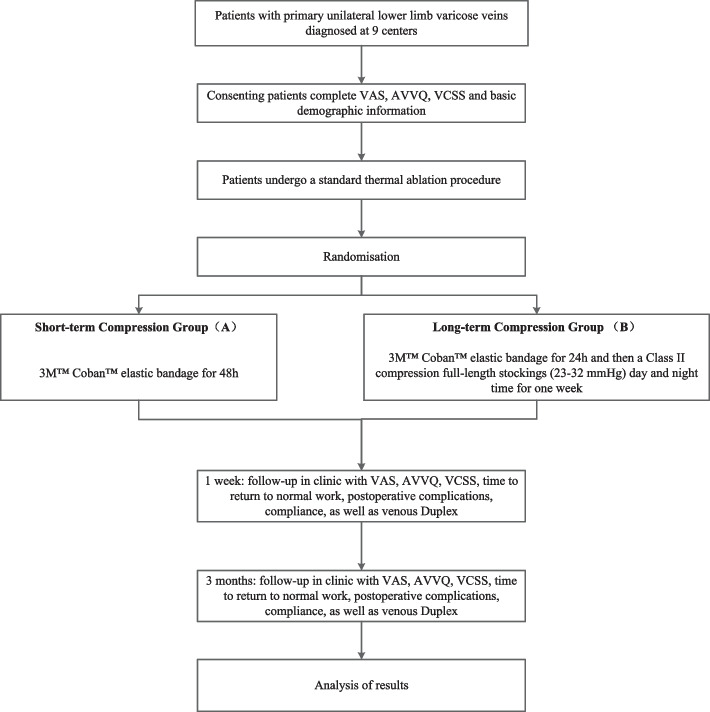
Trial flowchart. VAS, visual analog scale; AVVQ, Aberdeen Varicose Vein Questionnaire; VCSS, Venous Clinical Severity Score

Before compression therapy, all patients will undergo a standard thermal ablation procedure (RFA, Medtronic Closure Fast or EVLA, Eufoton 1470 nm ring laser). Patients will be randomized to groups A or B. Patients randomized to group A will be asked to wear the 3 M™ Coban™ elastic bandage for 48 h after the surgery. Patients randomized to group B will be asked to wear the 3 M™ Coban™ elastic bandage for 24 h and then a class II compression full-length stockings (23–32 mmHg) day and night time for 1 week (Fig. 1). The surgeon will place the bandages on the patient's leg while still lying on the operating table after thermal ablation. By the way, implementing short-term or long-term compression therapy will not require alteration to usual care pathways (including the use of any medication) and these will continue for both trial arms.

#### Primary outcome

The study’s primary outcome is the target vein occlusion rate at 3 months, assessed with a duplex ultrasound scan. Recanalization will be defined by a segment of the vein ≥ 5 cm.

Target vein occlusion rate = number of target venous closure cases in the group/total number of cases in the group.

#### Secondary outcomes

The secondary outcomes include the following:

- Pain
  Patients will be asked to record pain scores with VAS, and the scale ranges from 0 (no pain) to 10 (worst pain imaginable) [26].
- Quality of life
  Patients will be asked to fill out the AVVQ to measure the health status of varicose vein patients based on symptoms and impact on daily activities. A total score ranging from 0 to 35, with 35 being the worst quality of life [27].
- Venous clinical severity
  Patients will be asked to fill out the VCSS to evaluate the severity of the hallmarks of venous disease. The questionnaire consisted of ten questions, each of which was answered by 0 (none), 1 (mild), 2 (moderate), and 3 (severe)—a total score ranging from 0 to 30, with 30 being the worst quality of venous disease [28].
- Postoperative complications
  Postoperative complications include postoperative edema, ecchymosis, sensory abnormalities, skin burns/discoloration, phlebitis, hematoma, infection, deep vein thrombosis, and pulmonary embolism.
- Time to return to regular work
  The investigator will use a uniform question to ask the patient how long it takes to return to regular work, life, or both (days), and then record.
- Compliance
  Compliance refers to the extent to which patients follow compression therapy as recommended by their clinicians. Investigators will ask patients whether they have completed compression treatment as recommended by their doctors in the past week or 3 months. The answer to this question involves 1 (none), 2 (occasionally), 3 (mostly), and 4 (entirely). Those who still need to fully follow the study protocol for compression treatment should continue to be asked about their actual compression treatment and recorded.

#### Trial timescales

**Fig. 2:**
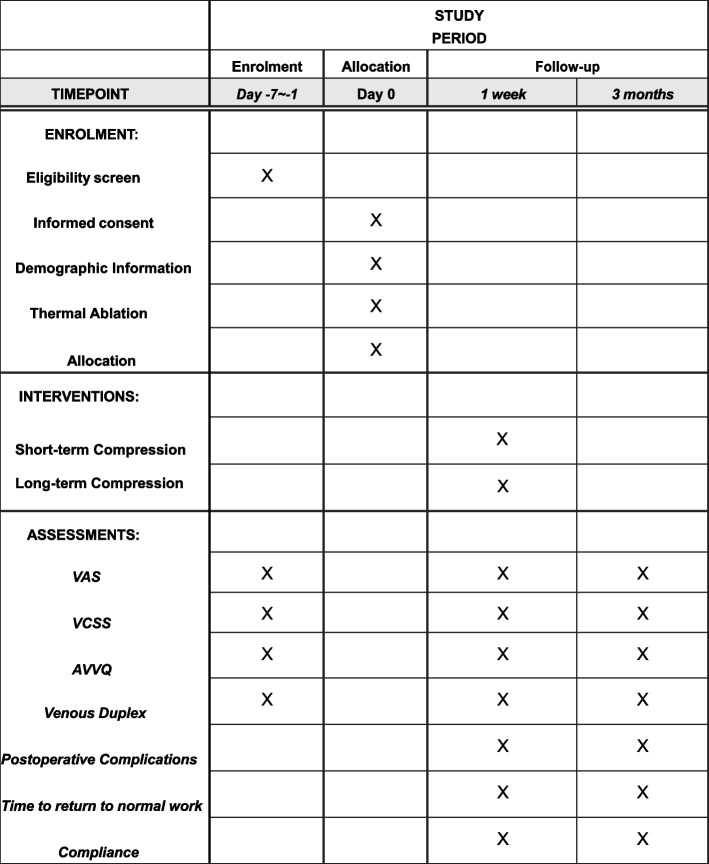
Standard Protocol Items: Recommendations for Interventional Trials (SPIRIT) flow sheet schedule of enrolment, interventions and assessments. VAS visual analogue scale, AVVQ Aberdeen Varicose Vein Questionnaire, VCSS Venous Clinical Severity Score

At the current rate of patients presenting to the centers, relatively broad inclusion criteria, and experience from previous trials of this nature, this study is expected to be completed within approximately twelve months (Fig. 2, Additional file 1. SPIRIT Checklist for Trials).

#### Baseline

Each center will have two permanent medical staff to gather information and conduct follow-ups, an investigator to collect information, and a checker to evaluate the quality of questionnaires and patient cooperation. All investigators will be trained before the official follow-up. At baseline, primary demographic data will be collected for each patient. Patients’ contact details will also be collected to enable the scheduling of follow-up appointments. Patients will be asked to record pain scores with VAS, fill out a quality of life questionnaire (AVVQ), and assess their clinical scores (CEAP and VCSS). They will also be asked to attend a follow-up in 1 week and at 3 months.

#### Follow-up

Patients will be followed up in the outpatient clinic at 1 week and 3 months. At the 1 week follow-up, patients will be asked to record pain scores with VAS, fill out a quality of life questionnaire (AVVQ), and will have their clinical scores assessed (VCSS). They will also be asked how soon they could return to regular work, postoperative complications, and compliance with the intervention. A venous Duplex scan will be held to determine the occlusion of the target vein. Follow-up at 3 months after thermal ablation will be the same as at 1 week follow-up.

#### Sample size and study duration

According to the literature [29–31] and clinical experts’ recommendations, the target vein occlusion rate in the control group (group B) was set to 98%, and a non-inferiority threshold δ of − 6% was taken [17]. With a power of 90% and 2.5% (unilateral) significance equivalence, a minimum of 230 patients (115 per group) will need to be recruited. However, considering a potential drop-out rate of 20%, combined with the low probability of adverse events in this trial, the total sample size was expanded to 360 patients. With 3 months follow-up, the study will run for 12 months.

#### Recruitment

Recruitment will occur in outpatient clinics at each center; patients who meet the inclusion criteria will be made aware of this study and provided with the appropriate information. Patients expressing an interest in participation will be offered an appointment for a screening visit with a study investigator/ a doctor. After taking a detailed medical history and a detailed duplex ultrasound examination, if the potential participant meets the criteria and is willing to proceed to enrolment in the trial, they will sign informed consent forms. Here the potential participant will be fully briefed on the trial process, the treatments, risks, economic compensation, and follow-up.

#### Statistical analysis

Continuous data will be compared by the Student *t*-test or Mann–Whitney *U* test for parametric and non-parametric data, as appropriate. Categorical data will be compared by the chi-square test or Fisher’s exact test, as appropriate. Considering the participants might crossover or be lost to follow-up, we will test non-inferiority using two analysis sets. One is the intention-to-treat set, considering all patients as randomized regardless of whether they received the randomized treatment, the other is the per-protocol set. We propose declaring short-term compression therapy non-inferior to long-term compression therapy, only if shown to be non-inferior using both the “intention-to-treat” and “per-protocol” analysis sets. *P* < 0.05 will be considered statistically significant, and all data will be analyzed using SAS 9.2 software.

#### Data collection and confidentiality

To promote participant retention and complete follow-up, we will add WeChat and record the mobile phone number of each participant, and investigators will phone contact or send a text message to remind the participants to follow up before each study visit. The investigators will contact participants who default a scheduled appointment to re-arrange another appointment within 1 week. In addition, we will ask each participant about their compliance with compression therapy (one of the secondary outcomes) to assess whether participants discontinue or deviate from intervention protocols. Participants will be allocated an individual trial identification number according to the order of inclusion. The anonymized trial data will be shared with other researchers to enable international prospective meta-analyses. Information will be entered by Epidata 3.1 in pairs to ensure its quality.

All information collected for this study, including electronic versions (stored on USB sticks) and paper questionnaires, will be securely stored in password cabinets at each center for at least 10 years. Data collected during the research will be kept strictly confidential and only accessed by members of the trial team (or individuals from the Sponsor organization or center sites where relevant to the trial).

#### Data monitoring, safety, and quality control

The ethics committee of each center will audit trial conduct as the circumstances may require. Trial Management Group will meet to review trial conduct per month. The Trial Steering Group and the independent Data Monitoring meet to review conduct throughout the trial period.

No significant adverse events are expected. An adverse event (AE) is an untoward medical occurrence in a patient or clinical study subject, which may or may not be caused by the investigational device. All such events, whether expected or not, should be recorded. A severe adverse event (SAE) is an untoward and unexpected medical occurrence or effect that results in death or is life-threatening, explicitly referring to an event in which the subject was at risk of death at the time of the event. It does not refer to an event that hypothetically might have caused death if it were more severe, requires hospitalization or prolongation of existing inpatients’ hospitalization, results in persistent or significant disability or incapacity, or results from a congenital anomaly or congenital disability. All AEs should be reported as following procedures.

An SAE form should be completed and sent by fax or email to the chief investigator within 24 h. All SAEs should be reported to the Research Ethical Committee where, in the opinion of the chief investigator, the event was “related” (i.e., resulted from the administration of any of the research procedures) and “unexpected” (i.e., an event that is not listed in the Protocol as an expected occurrence). Reports of related and unexpected SAEs should be submitted within 15 days of the chief investigator’s awareness of the event, using the NRES SAE form for non-IMP studies. Local investigators should report any SAEs as required by their Local Research Ethics Committee, sponsor, and/or Research and Development Office.

The investigator and sponsor will do their best to prevent possible injury caused by the design of this study. In case of any injury related to this study, the sponsor will bear the corresponding treatment costs and make compensation by national laws and regulations. If an adverse event caused by the investigational compression treatment causes harm, the clinical research institution and the investigator will provide timely and adequate treatment and management according to the routine clinical treatment.

#### Publication of data and reproducible research

The findings from this study will be presented locally within the hospitals, published in a peer-reviewed journal, and presented at national and international conferences. The datasets analyzed during the current study and statistical code are available from the corresponding author on reasonable request, as is the full protocol.

#### Availability of data and materials

All data analyses and manuscripts produced will be available on request.

#### Dropout and discontinuation

Patients may voluntarily withdraw from the study for other treatments or no reason, or if a significant safety event occurs and the investigator deems it necessary to stop the trial, the subject may withdraw. However, participants are not encouraged to withdraw from the study voluntarily, and we will be fully prepared for the possibility of adverse events.

#### Ethical approval and study registration

The study has been approved by the Ethical Committee of The Fourth Affiliated Hospital Zhejiang University, School of Medicine (Approval NO.: K2023047). Written, informed consent to participate will be obtained from all participants. The study has been registered at the ClinicalTrials.gov website (https://beta.clinicaltrials.gov/study/NCT05840991). The study will follow the recommendations for physicians involved in research on human subjects adopted by the 18th World Medical Assembly, Helsinki 1964, and later revisions.

#### Protocol amendments

Notifying sponsor and funder first, then the PI will notify the centers, and that a copy of the revised protocol will be sent to the PI to add to the Investigator Site File. Any deviations from the Protocol will be fully documented using a breach report form, and the protocol will be updated in the clinical trial registry.

### Discussion

Although elastic bandages and compression stockings have been widely used for compression therapy after thermal ablation of varicose veins, prolonged compression therapy harms patients’ quality of life and finance. With the development of relevant clinical trials, it has been shown that prolonged compression therapy has no significant clinical gain and may be an unnecessary adjunctive treatment after thermal ablation. The target vein occlusion rate is a crucial concern for vascular surgeons, and simplifying the current compression therapy while ensuring the closure of the treated vein will significantly improve the quality of life and compliance of patients after surgery, reduce their economic burden, and help patients return to everyday life as soon as possible. Suppose the effect of short-term compression therapy proves to be non-inferior to long-term compression therapy. In that case, this trial may contribute to future updates of clinical guidelines for compression therapy after thermal ablation of varicose veins.

### Trial status

Study Protocol Version 1.0, dated March 15, 2023. Recruitment is ongoing, the first patient was enrolled on May 22, 2023, and recruitment is estimated to be completed on August 31, 2023.

### Supplementary Information

**Additional file 1. **SPIRIT Checklist for *Trials.*

**Additional file 2. **

## Abbreviations

HL/S: High ligating/stripping
RFA: Radiofrequency ablation
EVLA: Endovenous laser ablation
UGFS: Ultrasound-guided foam sclerotherapy
ABI: Ankle-brachial index
CEAP: Clinical–Etiological–Anatomical–Pathophysiology
VAS: Visual analog scale
AVVQ: Aberdeen Varicose Vein Questionnaire
VCSS: Venous Clinical Severity Score
